# Butane-1,4-diammonium bis­(pyridine-2-carboxyl­ate) monohydrate

**DOI:** 10.1107/S1600536809031493

**Published:** 2009-08-15

**Authors:** Nam-Ho Kim, Kwang Ha

**Affiliations:** a School of Applied Chemical Engineering, the Research Institute of Catalysis, Chonnam National University, Gwangju 500-757, Republic of Korea

## Abstract

The asymmetric unit of the title compound, C_4_H_14_N_2_^2+^·2C_6_H_4_NO_2_^−^·H_2_O, consists of half of a doubly protonated tetra­methyl­enediammonium dication, a pyridine-2-carboxyl­ate anion and half of a solvent water mol­ecule; the dication is located on a centre of inversion and a twofold rotation axis passes through the O atom of the water mol­ecule. The carboxyl­ate group of the anion appears to be delocalized on the basis of the C—O bond lengths. In the crystal structure, the components are linked by inter­molecular N—H⋯O, N—H⋯N and O—H⋯O hydrogen bonds.

## Related literature

For the crystal structures of some butane-1,4-diammonium compounds, see: Natarajan & Cheetham (1997 ▶); Zheng *et al.* (1999 ▶); Sediri *et al.* (2002 ▶); Srinivasan *et al.* (2005 ▶); Lemmerer & Billing (2006 ▶); van Blerk & Kruger (2007 ▶, 2008 ▶); Jayasundera *et al.* (2008 ▶). For the structure of pyridine-2-carboxylic acid, see: Hamazaki *et al.* (1998 ▶). For a related hexane-1,6-diammonium compound, see: Kim & Ha (2009 ▶).
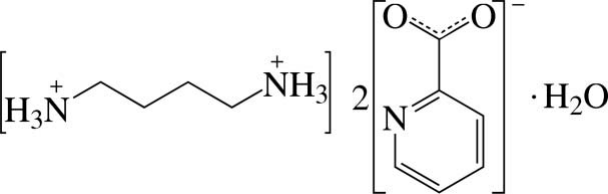


## Experimental

### 

#### Crystal data

C_4_H_14_N_2_^2+^·2C_6_H_4_NO_2_^−^·H_2_O
*M* *_r_* = 352.39
Monoclinic, 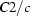

*a* = 20.655 (3) Å
*b* = 7.6170 (11) Å
*c* = 12.910 (2) Å
β = 113.789 (4)°
*V* = 1858.5 (5) Å^3^
*Z* = 4
Mo *K*α radiation
μ = 0.10 mm^−1^
*T* = 296 K
0.27 × 0.21 × 0.16 mm

#### Data collection

Bruker SMART 1000 CCD diffractometer
Absorption correction: multi-scan (**SADABS**; Bruker, 2000 ▶) *T*_min_ = 0.740, *T*_max_ = 0.985
6674 measured reflections
2298 independent reflections
1040 reflections with *I* > 2σ(*I*)
*R*_int_ = 0.048

#### Refinement

*R*[*F* ^2^ > 2σ(*F* ^2^)] = 0.056
*wR*(*F* ^2^) = 0.152
*S* = 0.99
2298 reflections
162 parameters
All H-atom parameters refined
Δρ_max_ = 0.18 e Å^−3^
Δρ_min_ = −0.16 e Å^−3^

### 

Data collection: *SMART* (Bruker, 2000 ▶); cell refinement: *SAINT* (Bruker, 2000 ▶); data reduction: *SAINT*; program(s) used to solve structure: *SHELXS97* (Sheldrick, 2008 ▶); program(s) used to refine structure: *SHELXL97* (Sheldrick, 2008 ▶); molecular graphics: *ORTEP-3* (Farrugia, 1997 ▶) and *PLATON* (Spek, 2009 ▶); software used to prepare material for publication: *SHELXL97*.

## Supplementary Material

Crystal structure: contains datablocks global, I. DOI: 10.1107/S1600536809031493/is2449sup1.cif

Structure factors: contains datablocks I. DOI: 10.1107/S1600536809031493/is2449Isup2.hkl

Additional supplementary materials:  crystallographic information; 3D view; checkCIF report

## Figures and Tables

**Table 1:** Hydrogen-bond geometry (Å, °)

*D*—H⋯*A*	*D*—H	H⋯*A*	*D*⋯*A*	*D*—H⋯*A*
N2—H2*A*⋯O2^i^	0.99 (3)	1.77 (3)	2.749 (3)	170 (2)
N2—H2*B*⋯O1^ii^	0.96 (3)	1.84 (3)	2.792 (3)	176 (2)
N2—H2*C*⋯O1^iii^	0.95 (3)	2.26 (3)	2.997 (3)	134 (2)
N2—H2*C*⋯N1^iii^	0.95 (3)	2.06 (3)	2.917 (3)	149 (2)
O3—H3*O*⋯O1^iv^	0.92 (4)	2.02 (4)	2.926 (3)	172 (4)

Symmetry codes: (i) 
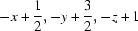
; (ii) 
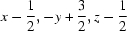
; (iii) 
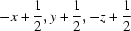
; (iv) 
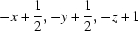
.

**Fig. 1.**
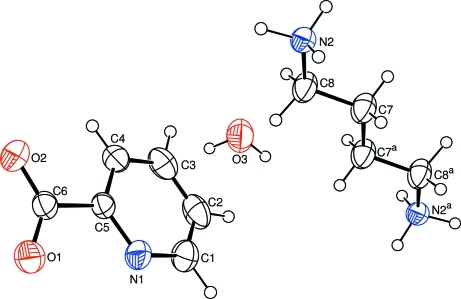
The structure of the title compound, with displacement ellipsoids drawn at the 40% probability level for non-H atoms. The superscript a corresponds to symmetry code: -x, -y + 1, -z.

**Fig. 2.**
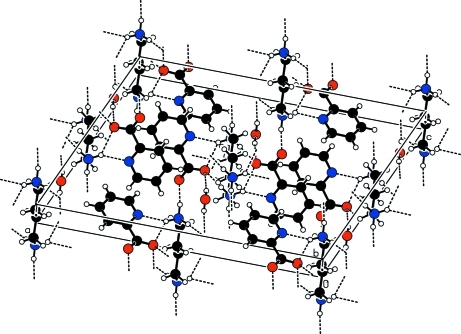
View of the unit-cell contents of the title compound. Hydrogen-bond interactions are drawn with dashed lines.

## supplementary crystallographic information

### Comment

The title compound, C_4_H_14_N_2_^2+^.2C_6_H_4_NO_2_^-^.H_2_O, consists of a doubly protonated tetramethylenediammonium dication, two pyridine-2-carboxylate anions and a solvent water molecule and the asymmetric unit contains one half of the formula unit (Fig. 1); a centre of inversion is located at the mid-point of the dication and the water molecule is disposed about a twofold rotation axis through O atom with the special position at (0, *y*, 1/4) (Wyckoff letter e). The carboxylate groups of the anions appear to be delocalized on the basis of the C—O bond lengths [C—O: 1.235 (3) and 1.251 (3) Å]. The torsion angles within the dication reveal that all N and C atoms of the dication display the anti conformation. In the crystal structure, the components are linked by intermolecular N—H···O, N—H···N and O—H···O hydrogen bonds (Table 1 and Fig. 2). There may also be intermolecular π–π interactions between adjacent pyridine rings, with a centroid-centroid distance of 3.796 (2) Å.

### Experimental

A solution of 1,4-diaminobutane (0.200 g, 2.269 mmol) and pyridine-2-carboxylic acid (1.173 g, 9.528 mmol) in H_2_O (20 ml) was stirred for 3 h at 60 °C. The solvent was removed under vacuum and the residue was washed with acetone/ether, to give a white powder (0.830 g). Crystals suitable for X-ray analysis were obtained by slow evaporation from an acetone solution.

### Refinement

All H atoms were located from Fourier difference maps and refined isotropically; C—H = 0.91 (2)–1.05 (3) Å, N—H = 0.95 (3)–0.99 (3) Å and O—H = 0.92 (4) Å.

### Crystal data

**Table:**

C_4_H_14_N_2_^2+^·2C_6_H_4_NO_2_^−^·H_2_O	*F*(000) = 752
*M**_r_* = 352.39	*D*_x_ = 1.259 Mg m^−^^3^
Monoclinic, *C*2/*c*	Mo *K*α radiation, λ = 0.71073 Å
Hall symbol: -C 2yc	Cell parameters from 1114 reflections
*a* = 20.655 (3) Å	θ = 2.9–22.5°
*b* = 7.6170 (11) Å	µ = 0.10 mm^−^^1^
*c* = 12.910 (2) Å	*T* = 296 K
β = 113.789 (4)°	Block, colorless
*V* = 1858.5 (5) Å^3^	0.27 × 0.21 × 0.16 mm
*Z* = 4	

### Data collection

**Table:**

Bruker SMART 1000 CCD diffractometer	2298 independent reflections
Radiation source: fine-focus sealed tube	1040 reflections with *I* > 2σ(*I*)
graphite	*R*_int_ = 0.048
φ and ω scans	θ_max_ = 28.3°, θ_min_ = 2.2°
Absorption correction: multi-scan (*SADABS*; Bruker, 2000)	*h* = −27→27
*T*_min_ = 0.740, *T*_max_ = 0.985	*k* = −10→9
6674 measured reflections	*l* = −17→15

### Refinement

**Table:**

Refinement on *F*^2^	Primary atom site location: structure-invariant direct methods
Least-squares matrix: full	Secondary atom site location: difference Fourier map
*R*[*F*^2^ > 2σ(*F*^2^)] = 0.056	Hydrogen site location: inferred from neighbouring sites
*wR*(*F*^2^) = 0.152	All H-atom parameters refined
*S* = 0.99	*w* = 1/[σ^2^(*F*_o_^2^) + (0.0576*P*)^2^] where *P* = (*F*_o_^2^ + 2*F*_c_^2^)/3
2298 reflections	(Δ/σ)_max_ < 0.001
162 parameters	Δρ_max_ = 0.18 e Å^−^^3^
0 restraints	Δρ_min_ = −0.16 e Å^−^^3^

### Special details

**Table:**

Geometry. All e.s.d.'s (except the e.s.d. in the dihedral angle between two l.s. planes) are estimated using the full covariance matrix. The cell e.s.d.'s are taken into account individually in the estimation of e.s.d.'s in distances, angles and torsion angles; correlations between e.s.d.'s in cell parameters are only used when they are defined by crystal symmetry. An approximate (isotropic) treatment of cell e.s.d.'s is used for estimating e.s.d.'s involving l.s. planes.
Refinement. Refinement of *F*^2^ against ALL reflections. The weighted *R*-factor *wR* and goodness of fit *S* are based on *F*^2^, conventional *R*-factors *R* are based on *F*, with *F* set to zero for negative *F*^2^. The threshold expression of *F*^2^ > σ(*F*^2^) is used only for calculating *R*-factors(gt) *etc*. and is not relevant to the choice of reflections for refinement. *R*-factors based on *F*^2^ are statistically about twice as large as those based on *F*, and *R*- factors based on ALL data will be even larger.

### Fractional atomic coordinates and isotropic or equivalent isotropic displacement parameters (Å^2^)

**Table:**

	*x*	*y*	*z*	*U*_iso_*/*U*_eq_	
O1	0.42900 (8)	0.4778 (2)	0.55250 (14)	0.0629 (6)	
O2	0.36511 (9)	0.6362 (3)	0.61996 (15)	0.0793 (7)	
N1	0.31266 (9)	0.3696 (3)	0.37672 (16)	0.0541 (6)	
C1	0.25453 (15)	0.3005 (4)	0.2959 (2)	0.0663 (8)	
H1	0.2608 (13)	0.230 (4)	0.235 (2)	0.089 (9)*	
C2	0.18842 (15)	0.3185 (4)	0.2948 (3)	0.0685 (8)	
H2	0.1484 (13)	0.262 (3)	0.233 (2)	0.073 (8)*	
C3	0.18017 (14)	0.4148 (4)	0.3778 (3)	0.0646 (8)	
H3	0.1342 (14)	0.439 (3)	0.380 (2)	0.085 (9)*	
C4	0.23908 (13)	0.4901 (4)	0.4606 (2)	0.0547 (7)	
H4	0.2350 (11)	0.556 (3)	0.517 (2)	0.055 (7)*	
C5	0.30461 (11)	0.4623 (3)	0.45825 (18)	0.0429 (6)	
C6	0.37158 (13)	0.5323 (3)	0.5513 (2)	0.0483 (6)	
N2	0.05485 (12)	0.8325 (3)	0.15154 (19)	0.0463 (5)	
H2A	0.0797 (12)	0.853 (3)	0.234 (2)	0.072 (8)*	
H2B	0.0116 (14)	0.898 (3)	0.121 (2)	0.070 (8)*	
H2C	0.0864 (14)	0.863 (4)	0.117 (2)	0.091 (10)*	
C7	0.00642 (18)	0.5974 (3)	0.0102 (2)	0.0599 (8)	
H7A	−0.0362 (16)	0.665 (4)	−0.025 (3)	0.106 (11)*	
H7B	0.0374 (16)	0.642 (4)	−0.025 (3)	0.116 (12)*	
C8	0.04043 (18)	0.6425 (4)	0.1318 (2)	0.0612 (8)	
H8A	0.0144 (16)	0.604 (4)	0.173 (3)	0.113 (13)*	
H8B	0.0889 (17)	0.577 (4)	0.175 (3)	0.127 (13)*	
O3	0.0000	0.2467 (4)	0.2500	0.0872 (10)	
H3O	0.018 (2)	0.173 (5)	0.311 (3)	0.176 (18)*	

### Atomic displacement parameters (Å^2^)

**Table:**

	*U*^11^	*U*^22^	*U*^33^	*U*^12^	*U*^13^	*U*^23^
O1	0.0443 (9)	0.0705 (12)	0.0630 (12)	−0.0030 (9)	0.0102 (8)	−0.0125 (9)
O2	0.0782 (13)	0.0863 (15)	0.0542 (12)	0.0161 (11)	0.0066 (10)	−0.0263 (11)
N1	0.0472 (12)	0.0625 (14)	0.0459 (12)	−0.0017 (10)	0.0117 (10)	−0.0095 (10)
C1	0.0599 (18)	0.0692 (19)	0.0532 (17)	−0.0025 (15)	0.0056 (14)	−0.0149 (14)
C2	0.0483 (17)	0.0638 (19)	0.068 (2)	−0.0074 (14)	−0.0032 (14)	0.0020 (15)
C3	0.0429 (16)	0.073 (2)	0.071 (2)	0.0037 (14)	0.0156 (15)	0.0136 (16)
C4	0.0538 (16)	0.0597 (18)	0.0500 (16)	0.0119 (13)	0.0202 (13)	0.0062 (13)
C5	0.0462 (13)	0.0407 (13)	0.0368 (13)	0.0041 (11)	0.0116 (10)	0.0035 (10)
C6	0.0527 (14)	0.0423 (14)	0.0412 (14)	0.0048 (12)	0.0097 (12)	0.0015 (11)
N2	0.0456 (12)	0.0466 (13)	0.0425 (13)	0.0015 (10)	0.0134 (11)	−0.0049 (10)
C7	0.082 (2)	0.0495 (16)	0.0436 (15)	−0.0082 (16)	0.0205 (15)	−0.0045 (12)
C8	0.089 (2)	0.0471 (17)	0.0409 (15)	−0.0074 (15)	0.0191 (15)	−0.0047 (12)
O3	0.108 (2)	0.072 (2)	0.063 (2)	0.000	0.0152 (18)	0.000

### Geometric parameters (Å, °)

**Table:**

O1—C6	1.251 (3)	C5—C6	1.517 (3)
O2—C6	1.235 (3)	N2—C8	1.479 (3)
N1—C5	1.332 (3)	N2—H2A	0.99 (3)
N1—C1	1.341 (3)	N2—H2B	0.96 (3)
C1—C2	1.367 (4)	N2—H2C	0.95 (3)
C1—H1	1.01 (3)	C7—C8	1.478 (3)
C2—C3	1.364 (4)	C7—C7^i^	1.512 (5)
C2—H2	0.98 (2)	C7—H7A	0.96 (3)
C3—C4	1.380 (4)	C7—H7B	0.98 (3)
C3—H3	0.98 (3)	C8—H8A	0.95 (3)
C4—C5	1.383 (3)	C8—H8B	1.05 (3)
C4—H4	0.91 (2)	O3—H3O	0.92 (4)
			
C5—N1—C1	117.7 (2)	C8—N2—H2A	108.6 (15)
N1—C1—C2	123.1 (3)	C8—N2—H2B	110.4 (14)
N1—C1—H1	117.5 (15)	H2A—N2—H2B	111 (2)
C2—C1—H1	119.4 (15)	C8—N2—H2C	106.6 (17)
C3—C2—C1	119.1 (3)	H2A—N2—H2C	108 (2)
C3—C2—H2	122.5 (15)	H2B—N2—H2C	112 (2)
C1—C2—H2	118.4 (15)	C8—C7—C7^i^	112.8 (3)
C2—C3—C4	118.8 (3)	C8—C7—H7A	109.3 (19)
C2—C3—H3	123.6 (16)	C7^i^—C7—H7A	112.5 (19)
C4—C3—H3	117.5 (16)	C8—C7—H7B	106.8 (19)
C3—C4—C5	119.0 (3)	C7^i^—C7—H7B	111.1 (19)
C3—C4—H4	120.6 (14)	H7A—C7—H7B	104 (2)
C5—C4—H4	120.4 (14)	C7—C8—N2	112.7 (2)
N1—C5—C4	122.3 (2)	C7—C8—H8A	113.3 (19)
N1—C5—C6	116.6 (2)	N2—C8—H8A	108.9 (19)
C4—C5—C6	121.1 (2)	C7—C8—H8B	113.5 (17)
O2—C6—O1	125.5 (2)	N2—C8—H8B	106.6 (17)
O2—C6—C5	117.7 (2)	H8A—C8—H8B	101 (3)
O1—C6—C5	116.8 (2)		
			
C5—N1—C1—C2	−1.1 (4)	C3—C4—C5—C6	−176.1 (2)
N1—C1—C2—C3	1.7 (5)	N1—C5—C6—O2	171.3 (2)
C1—C2—C3—C4	−0.4 (4)	C4—C5—C6—O2	−10.4 (3)
C2—C3—C4—C5	−1.4 (4)	N1—C5—C6—O1	−9.5 (3)
C1—N1—C5—C4	−0.8 (4)	C4—C5—C6—O1	168.8 (2)
C1—N1—C5—C6	177.5 (2)	C7^i^—C7—C8—N2	178.3 (3)
C3—C4—C5—N1	2.1 (4)		

Symmetry codes: (i) −*x*, −*y*+1, −*z*.

### Hydrogen-bond geometry (Å, °)

**Table:**

*D*—H···*A*	*D*—H	H···*A*	*D*···*A*	*D*—H···*A*
N2—H2A···O2^ii^	0.99 (3)	1.77 (3)	2.749 (3)	170 (2)
N2—H2B···O1^iii^	0.96 (3)	1.84 (3)	2.792 (3)	176 (2)
N2—H2C···O1^iv^	0.95 (3)	2.26 (3)	2.997 (3)	134 (2)
N2—H2C···N1^iv^	0.95 (3)	2.06 (3)	2.917 (3)	149 (2)
O3—H3O···O1^v^	0.92 (4)	2.02 (4)	2.926 (3)	172 (4)

Symmetry codes: (ii) −*x*+1/2, −*y*+3/2, −*z*+1; (iii) *x*−1/2, −*y*+3/2, *z*−1/2; (iv) −*x*+1/2, *y*+1/2, −*z*+1/2; (v) −*x*+1/2, −*y*+1/2, −*z*+1.
